# Obstructive Sleep Apnea and Cardiovascular Disease: Mechanisms, Diagnostics, and Emerging Therapeutic Approaches

**DOI:** 10.3390/biomedicines14061263

**Published:** 2026-06-01

**Authors:** Bridget R. Alber, Emily C. Cheung, Rebekah Russo, Vivek Jain, Kathryn Jaques Schunke, David Mendelowitz, Matthew W. Kay

**Affiliations:** 1 Department of Biomedical Engineering, The George Washington University, Washington, DC 20052, USA; balber@gwmail.gwu.edu (B.R.A.); eccheung@gwmail.gwu.edu (E.C.C.); bekahdrusso@gwmail.gwu.edu (R.R.); 2 Division of Pulmonary, Critical Care & Sleep Medicine, Department of Medicine, The George Washington University, Washington, DC 20037, USA; vjain@mfa.gwu.edu; 3 Department of Cell and Molecular Biology, University of Hawaii, Honolulu, HI 96813, USA; kschunke@hawaii.edu; 4 Department of Pharmacology and Physiology, The George Washington University, Washington, DC 20052, USA

**Keywords:** obstructive sleep apnea, central sleep apnea, continuous positive airway pressure, cardiovascular disease, precision medicine, nerve stimulation, epigenetics

## Abstract

Current diagnostic methods for OSA and CSA are costly, unreliable, and the therapeutic options are limited, with varying efficacy across patients. As the prevalence of sleep apnea and cardiovascular disease continue to rise, identifying innovative therapies through advances in biomedicine and personalized medicine has become increasingly critical. This review discusses the mechanistic links between sleep apnea and subsequent cardiovascular outcomes from a technical perspective, focusing on innovations currently applied to the diagnosis and treatment of sleep apnea, and opportunities for further advancement in the field.

## 1. Introduction

Sleep apnea, a form of sleep disordered breathing, is a major public health issue that significantly heightens the risk for cardiovascular disease (CVD) and mortality. There are two primary forms of sleep apnea, obstructive sleep apnea (OSA) and central sleep apnea (CSA). Both are characterized by distinct underlying mechanisms, but remain prevalent in the general population, contributing to poor sleep quality, diminished quality of life, and detrimental cardiovascular effects. Autonomic imbalance induced by sleep apnea can trigger life-threatening arrhythmias and contribute to cardiovascular mortality. Additionally, reactive oxygen species (ROS) generated during hypoxic events promote atherosclerotic progression, increasing the risk of myocardial infarction and stroke and accelerating the development of heart failure (HF). As the prevalence of sleep apnea and cardiovascular disease continue to rise, identifying innovative therapies through advances in biomedicine and personalized medicine has become increasingly critical. This review discusses the mechanistic links between sleep apnea and subsequent cardiovascular outcomes from a technical perspective, focusing on innovations currently applied to the diagnosis and treatment of sleep apnea, and opportunities for further advancement in the field.

## 2. OSA vs. CSA

**Figure 1:**
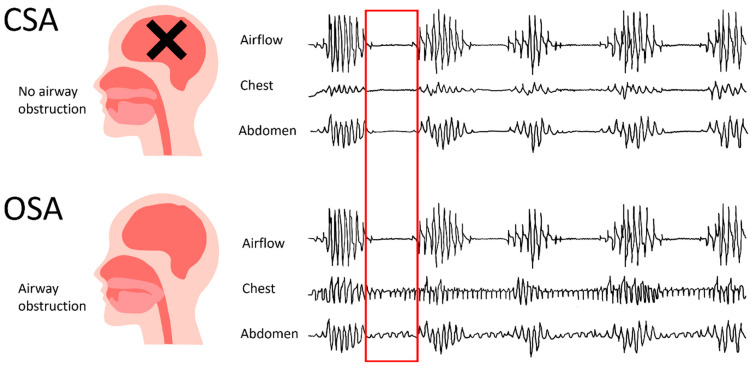
Comparison of central sleep apnea (CSA) and obstructive sleep apnea (OSA) patterns. The diagram illustrates idealized differences in airflow and respiratory effort during apneic events. In CSA, there is no airway obstruction, and both chest and abdominal movements cease, indicating a lack of respiratory effort. OSA is characterized by airway obstruction with continued respiratory effort observed in the chest and abdomen without airflow. The highlighted section (red box) shows the absence of airflow in both conditions, with different respiratory efforts.

OSA is characterized by reduced airflow due to repetitive upper airway obstructions during sleep [1], while CSA results from recurrent attenuation or cessation of respiratory reflex effort [2], leading to reduced or absent ventilation [3] (Figure 1). Both produce abnormally low levels of oxygen and elevated CO_2_ in the body. These disruptions cause frequent arousals from sleep, resulting in poor sleep quality, daytime sleepiness, and diminished quality of life.

OSA severity is determined by the number of apneas (complete obstructions) and hypopneas (partial obstructions) per hour during sleep, quantified as an apnea-hypopnea index (AHI) [4]. Mild OSA is characterized by an AHI between 5 and 15 events per hour, moderate OSA falls within an AHI range between 15 and 30 events per hour, and severe OSA presents with an AHI surpassing 30 events per hour [5]. While prevalence of OSA varies across populations, it is estimated to affect approximately 3–7% of men and 2–5% of women [6]. OSA is more prevalent in Hispanic, Black and Asian populations, and risk factors include obesity and advanced age [7], with overweight males being at the highest risk [8]. In contrast, CSA is considerably less prevalent, affecting an estimated 1.8% of middle-aged adults [9]. This prevalence increases to 7.5% for adults over 65 years of age [3].

Research over the past decade has demonstrated strong associations between CSA and both cardiovascular disorders and chronic opioid use [10,11]. The clinical picture is complicated by numerous comorbidities associated with both forms of sleep apnea. OSA commonly presents alongside age-related changes, obesity, hypertension, smoking, and alcohol use [1], while CSA risk factors typically include advanced age, male sex, and HF. Complex sleep apnea, characterized by the coexistence of both OSA and CSA, can further complicate diagnosis and disease understanding [2].

### 2.1. Pathophysiological Mechanisms

**Figure 2:**
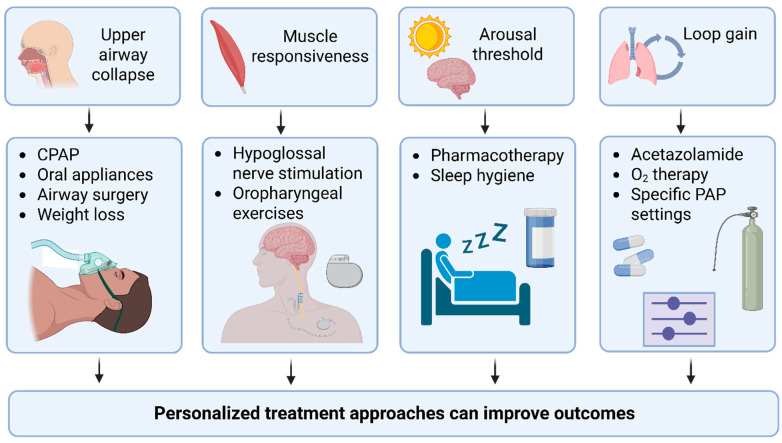
Mechanistic framework for obstructive sleep apnea and therapeutic targets. OSA arises from four overlapping endotypes: elevated upper airway collapsibility, impaired pharyngeal muscle responsiveness, low arousal threshold, and high loop gain. The effectiveness of targeted interventions including CPAP, hypoglossal nerve stimulation, and pharmacologic modulation may depend on the underlying endotype. Endotype-based phenotyping may help guide personalized OSA treatment strategies and improve therapeutic effectiveness. Created in BioRender. Kay, M. (2026) https://BioRender.com/rjvaf2n, accessed on 19 February 2026.

While the etiologies of OSA and CSA vary significantly among patients, both conditions share similar pathophysiological consequences (Figure 2). OSA originates from the collapse of the upper airway between the hard palate and larynx during sleep [12]. Intramuscular electromyography (EMG) recordings of the genioglossus, the largest upper airway dilator muscle in humans, reveal that both healthy individuals and those with OSA experience diminished upper airway muscle tone at sleep onset. However, individuals with anatomic vulnerability in the upper airway who rely more heavily on muscle tone to maintain airway patency are more susceptible to developing OSA [13]. Additionally, OSA patients lack stability in ventilatory control, leading to oscillations between obstructive events and arousals. Ventilatory control stability can be explained through the concept of loop gain [14] where chemoreceptor responsiveness to hypoxia or hypercapnia and the efficiency of CO_2_ excretion from the system are pivotal factors in the ventilatory feedback control system. Loop gain, mathematically within the ventilation context, is described as the ratio of the ventilatory response to the ventilatory disturbance, where higher loop gain reflects a more unstable breathing control system. It determines whether breathing oscillations lead to apneas [15].

In CSA, elevated loop gain arises from dysregulation of the negative feedback control system. During normal breathing, changes in ventilatory effort alter partial pressure of CO_2_ (PCO2) in the lungs and arteries, which chemoreceptors sense and use to adjust ventilation until equilibrium is achieved. In CSA, periods of hyperpnea and arousal cause PCO2 to drop below normal levels. When this hypocapnic blood reaches chemoreceptors, ventilatory effort decreases. However, time delays between the PCO2 drop and chemoreceptor response create a ventilatory undershoot, where ventilation falls below optimal levels, triggering a rise in arterial PCO2, prompting a reflexive ventilatory overshoot and perpetuating a cycle of arousal followed by hypoventilation [16]. The mechanism contributes to Cheyne–Stokes respiration observed in patients with HF, where heightened chemoreflex sensitivity, reduced CO2 reserve, and prolonged circulatory delay can further elevate loop gain [17]. Pharmacologic strategies that lower loop gain have been explored as approaches to widen the CO2 reserve, stabilize ventilatory control, and reduce central apnea burden in some patients [18].

Frequent arousals induced by ventilatory instability are particularly detrimental during sleep, which normally serves as a restorative phase for multiple organ systems, including the cardiovascular system. During healthy sleep, heart rate (HR) and blood pressure (BP) decline due to increased parasympathetic tone and decreased sympathetic activity, resulting in reduced HR, BP, stroke volume, and systemic vascular resistance [19,20]. In contrast, arousals from sleep trigger heightened sympathetic activity and diminished cardiac vagal activity that exceed normal waking levels, causing significant surges in HR and BP [21]. In patients with OSA, recurrent respiratory events and associated arousals disrupt sympatho-vagal balance during sleep and repetitive episodes of apnea and hypoxia activate the chemoreceptor reflex, further increasing sympathetic activation [22]. This chronic sympathetic activation contributes to arrhythmia, HF, hypertension, and sudden cardiac death [23].

### 2.2. Diagnosis of Sleep Apnea

The gold standard for diagnosing OSA and CSA is an overnight polysomnography (PSG), a comprehensive assessment that includes electroencephalogram, electrooculogram, electrocardiogram, electromyogram, nasal and oral airflow measurements, respiratory movement (thorax and abdominal respiratory effort), snoring detection, oxygen saturation, body position, and sleep staging [24]. Within PSG, respiratory inductance plethysmography (RIP) plays a crucial role in distinguishing between OSA and CSA by capturing patterns of chest and abdomen movement. Thoracoabdominal motion detected by RIP indicates ongoing respiratory effort, signifying OSA, while the absence of motion indicates CSA (Figure with CSA vs. OSA).

Despite its diagnostic accuracy, PSG has significant limitations related to the recording environment. Patients are required to spend the night in a sleep laboratory, which may compromise sleep quality because of the unfamiliar setting and discomfort from multiple sensors. Additionally, sensor displacement during sleep can cause data loss and artifacts, reducing diagnostic reliability. Thermal imaging offers a promising noninvasive alternative for detecting and monitoring sleep apnea [25]. Thermal infrared imaging techniques can measure cardiac pulse without physical contact, potentially improving patient comfort and sleep quality during PSG [26,27]. This technology is also being studied as a noninvasive method for measuring airflow during PSG [28,29]. By reducing the number of attached sensors, these noncontact measurement approaches allow patients to sleep more naturally during diagnostic studies, leading to more clinically relevant data.

## 3. Sleep Apnea & Cardiovascular Disease

**Figure 3:**
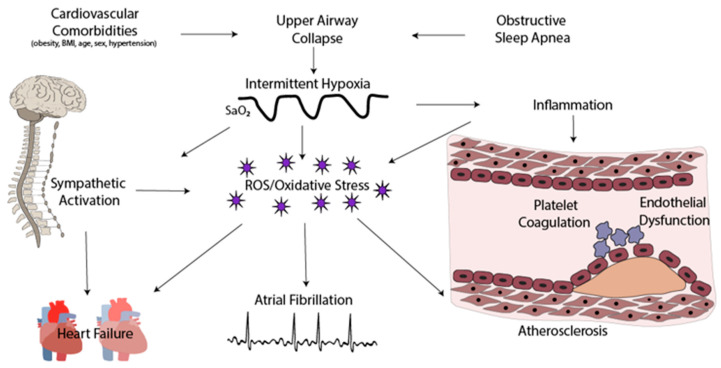
Pathophysiological mechanisms linking obstructive sleep apnea (OSA) to cardiovascular disease. The diagram illustrates how upper airway collapse in OSA leads to intermittent hypoxia, triggering a cascade of physiological responses including increased sympathetic activation, oxidative stress (ROS), and inflammation, contributing to endothelial dysfunction, platelet coagulation, and atherosclerosis. These responses increase the risk of cardiovascular comorbidities such as heart failure and atrial fibrillation.

Clinical and experimental evidence demonstrates that chronic intermittent hypoxia (CIH), a characteristic feature of sleep apnea, significantly contributes to multiple adverse cardiovascular complications through multiple pathophysiological mechanisms (Figure 3). The intermittent hypoxia leads to oxidative stress and sympathetic activation, which contribute to complications including endothelial dysfunction, accelerated atherosclerosis, myocardial ischemia, cardiac arrhythmias, and HF, imposing immediate and long-term morbidities, resulting in substantial healthcare costs [30,31,32].

### 3.1. Reactive Oxygen Species

While the precise mechanisms are not fully understood, repetitive hypoxia-reoxygenation cycles are thought to reduce oxygen tension in the blood, leading to the generation of ROS—a major driver of cardiovascular complications in sleep apnea. ROS activate key transcription factors including nuclear factor kappa B (NF-κB) and hypoxia-inducible factor-1 (HIF-1) [33], which modulate adaptive responses to hypoxia such as metabolic reprogramming, angiogenesis, and cell survival [34,35]. A recent study in mice found that HIF-1 activation was critical for eliciting the CIH-induced carotid body-mediated cardio-respiratory response [36]. CIH also elevated ROS levels, and the effects of CIH involved intricate and positive interactions between HIF-1 and ROS [36]. These findings illuminate the intricate molecular pathways underlying CIH-induced cardiovascular complications and may guide therapeutic interventions.

While predominantly an adaptive response, prolonged stabilization of HIF-1⍺ can gradually threaten cellular integrity and function. Among the many HIF-1 target genes, endothelin (ET-1) stands out for its vasoconstrictive, growth-promoting, and pro-inflammatory properties. Elevated levels of ET-1 have been observed in both OSA patients with hypertension and in animals exposed to CIH. ET-1 actively participates in pathophysiological pathways associated with adverse OSA outcomes, including hyperglycemia, myocardial infarction, HF, and stroke [37,38]. Recent studies demonstrated that cardiac-specific, inducible expression of oxygen-stable HIF-1⍺ in mice causes dilated blood vessels, enlarged hearts, and reduced ventricular function after 7 days of constitutive expression [39].

Conversely, in ischemic or hypoxic cardiomyopathies such as myocardial infarction, HIF-1 stabilization provides protection against functional decline by fostering angiogenesis, facilitating beneficial myocardial remodeling, and promoting miRNA-induced isoform switching that alters cardiovascular metabolism favorably [40]. These findings underscore the dual nature of HIF-1⍺ stabilization, highlighting its context-dependent impact on cellular dynamics and its pivotal role in shaping cardiovascular outcomes.

Endothelial dysfunction is a distinctive feature of OSA that worsens with disease severity [41]. Excessive ROS generation is a primary instigator of endothelial dysfunction, prompting vascular damage and accelerating atherosclerotic progression. Endothelial cell activation marks the onset of injury, and is characterized by a pro-inflammatory endothelial cell phenotype that reduces nitric oxide bioavailability and impairs vascular tone [42]. This heightened inflammation promotes smooth muscle proliferation and migration, increased intima-media thickness, and platelet activation with a pro-thrombotic phenotype. Together, these characteristics of ROS-induced endothelial dysfunction significantly increase the risk of acute vascular occlusions, myocardial infarction, and stroke. Several studies confirm that the progression of atherosclerosis in OSA is promoted by comorbidities such as obesity, body mass index, AHI, and age, with some suggesting OSA as an independent predictor of atherosclerosis, even after adjusting for confounding factors [43].

The landscape of evidence is evolving, as highlighted by a recent prospective clinical study (Epigenetic modifications in Obstructive Sleep Apnea, EPIOSA), which suggests that OSA may not independently predict atherosclerosis [44]. The mechanistic link between clinical OSA and increased ROS remains contested, with ongoing research exploring this complex association [45,46,47,48,49,50,51,52].

In summary, sleep apnea-induced intermittent hypoxia stimulates the production of reactive oxygen species (ROS), which activate transcriptional and inflammatory pathways (Figure 3). These responses can aid in adaptive cardio-respiratory functions but may also lead to endothelial dysfunction, vasoconstriction, and detrimental cardiovascular remodeling. Overall, the effects of these processes depend on the context—being protective in acute situations but possibly harmful when chronically activated—and their direct role in atherosclerosis in OSA is still a subject of debate.

### 3.2. Cardiac Arrhythmias

OSA is strongly associated with various life-threatening cardiac arrhythmias and plays a contributory role in their development [53]. The link is particularly robust for atrial fibrillation (AF), as a substantial proportion of AF patients also present with OSA [54]. Sleep apnea severity, measured in AHI, demonstrates a positive correlation with arrhythmia prevalence [55].

While comorbidities such as obesity have traditionally been implicated in cardiac arrhythmia development, studies indicate that OSA and obesity are independent risk factors for AF [56]. The severity of sleep-disordered breathing has been associated with AF prevalence [57], potentially through mechanisms including intermittent hypoxia, arterial pressure fluctuations, and sympathetic nervous system activation. Animal models of OSA have provided further mechanistic insight into these clinical observations. In animal models, CIH exposure reproduces the sympatho-vagal imbalance and atrial remodeling that is seen in OSA patients and are risk factors for AF [58]. A recent study of the autonomic nervous system and OSA revealed that both intrinsic and extrinsic cardiac autonomic factors significantly contribute to OSA-induced AF [59]. Hyperactivity in these systems was identified as a key mechanism, and experiments using vagal nerve stimulation to restore sympatho-vagal balance showed promise in preventing OSA-induced AF [59].

OSA is also strongly linked to recurrent AF [56]. Patients with OSA display atrial remodeling including atrial enlargement, conduction abnormalities, and prolonged sinus node recovery time [60]. These structural and electrical changes provide mechanistic insights into the relationship between AF and OSA, underscoring the importance of comprehensive management strategies. Improved diagnostic approaches and targeted OSA treatments may therefore reduce the risk of life-threatening arrhythmias in affected patients.

### 3.3. Heart Failure

CSA commonly occurs in patients with severe HF and is often considered a consequence of advanced HF, though it rarely develops in the absence of underlying disorders. The mechanisms underlying the CSA-HF association remain incompletely understood. Some evidence suggests that CSA may function as a compensatory mechanism in HF, though this remains debated.

A characteristic respiratory pattern observed in CSA patients with HF is Cheyne–Stokes respiration, characterized by cyclical oscillations between apnea and hyperventilation during sleep [61]. This distinctive respiration pattern is observed in 30–40% of individuals with congestive HF and systolic dysfunction [2], and in 5–10% of patients with sleep apnea [62]. While Cheyne–Stokes respiration causes hypoxic intervals and autonomic dysregulation, some argue that these detriments could be outweighed by the advantages of increased expiratory lung volume, positive airway pressure generation, and sympathetic nervous system inhibition [63]. This paradox highlights the complex relationship between CSA and HF, where respiratory instability may simultaneously contribute to disease progression and provide compensatory support.

## 4. Therapeutic Approaches

Sleep apnea treatments aim to normalize breathing patterns during sleep, alleviating symptoms and reducing associated cardiovascular complications. However, patient adherence remains a challenge, limiting therapeutic effectiveness. Ongoing research aims to improve upon current gold-standard therapies to enhance clinical outcomes and improve adherence to treatment.

### 4.1. OSA Therapies

**Figure 4:**
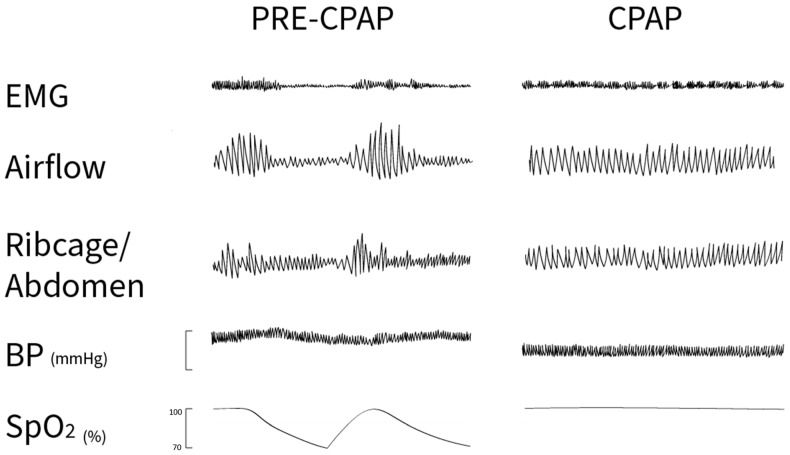
Effects of continuous positive airway pressure (CPAP) therapy on sleep apnea parameters. The figure compares representative idealized physiological recordings before and after CPAP treatment. Pre-CPAP shows irregular airflow, ribcage/abdominal movements, fluctuating blood pressure (BP), and decreased oxygen saturation (SpO2). Post-CPAP demonstrates stabilized airflow, consistent ribcage/abdominal movements, normalized BP, and improved SpO2 levels, indicating effective management of sleep apnea symptoms.

The current gold standard for managing OSA is Continuous Positive Airway Pressure (CPAP), which maintains airway patency during sleep (Figure 4). While CPAP effectively alleviates OSA symptoms, including excessive daytime sleepiness, cognitive dysfunction [64,65], and quality of life [66], its impact on OSA-related cardiovascular comorbidities remains less clear. Recent studies demonstrate that CPAP therapy significantly improves cognitive and psychomotor performance in OSA patients, with sleep quality and evening salivary cortisol levels closely linked to overall clinical outcomes [67,68]. In a randomized study of male OSA patients, CPAP reduced both systolic and diastolic blood pressure by 2–3 mmHg [69] and individuals with resistant hypertension exhibited improvements in diastolic BP with CPAP treatment [70]. However, a study of OSA patients without daytime sleepiness found that CPAP, compared to usual care, did not significantly reduce the incidence of hypertension or cardiovascular events (HR: 0.83, 95% CI: 0.63–1.1, *p* = 0.20) [71]. An adherence analysis revealed that patients using CPAP for at least 4 h per night showed a lower CVD risk (HR: 0.72, 95% CI: 0.52–0.98, *p* = 0.04). In patients with coronary artery disease, the Continuous Positive Airway Pressure Treatment of OSA to Prevent Cardiovascular Disease (SAVE) trial, involving 2602 individuals with a history of CVD and without moderate sleepiness who received either CPAP or usual care, found no benefit in cause-specific cardiovascular outcomes after a mean follow-up of 3.7 years (CPAP vs. usual care, HR: 1.10, 95% CI: 0.91–1.32, *p* = 0.34), despite improved sleepiness scores and quality-of-life measures [66].

These findings highlight the distinction between a benefit in symptoms and improvements in cardiovascular outcomes. Greater nightly adherence has been associated with lower cardiovascular risk, but this finding should be interpreted cautiously because adherence may also reflect differences in health behaviors, comorbidity burden, or access to care. Overall, CPAP improves symptoms and quality of life, but its ability to meaningfully modify cardiovascular risk in OSA remains a topic of debate.

A significant challenge in the field of OSA therapy is poor acceptance and adherence to CPAP treatment. Despite widespread availability of diagnostic and treatment services, insufficient compliance remains a major concern and contributes to continued disease burden. This suboptimal adherence not only hampers effective OSA management but also limits comprehensive understanding of trials investigating OSA’s impact on cardiovascular risk and the potential role of CPAP in mitigating that risk.

**Figure 5:**
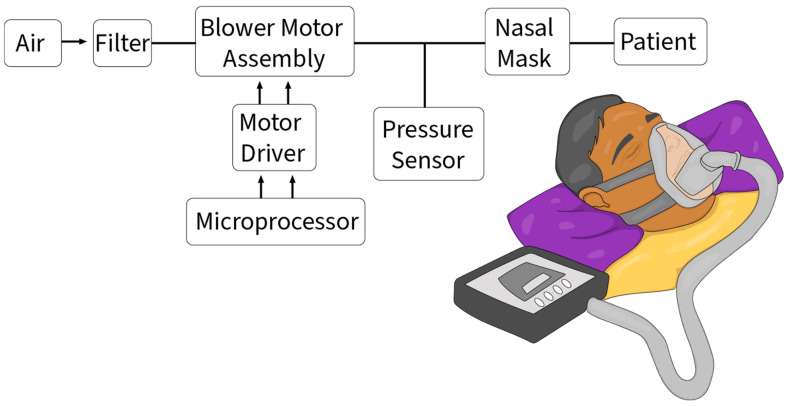
Diagram of a typical continuous positive airway pressure (CPAP) system. The illustration depicts the components of a CPAP device, including the air filter, blower motor assembly, motor driver, microprocessor, and pressure sensor. Air is filtered and pressurized before being delivered through a nasal mask to the patient, maintaining an open airway during sleep.

Several factors contribute to poor CPAP adherence. CPAP units are large, bulky, and often perceived as loud. These devices incorporate a water reservoir for humidity control, housing with a blower motor, motor driver, microprocessor, and pressure sensor, all connected via tubing to a nasal mask that forms a tight seal around the patient’s face to deliver positive pressure and prevent airway collapse during sleep [72] (Figure 5). Development of less cumbersome and less invasive CPAP-like devices has the potential to significantly enhance patient experience, increase adherence, and ultimately improve clinical outcomes.

A distinctive advantage of CPAP therapy is its capacity to reduce platelet reactivity, a precursor to thrombotic events. OSA is recognized as a risk factor for acute coronary syndrome, with studies demonstrating its association with increased platelet reactivity [73]. Multiple investigations indicate a correlation between platelet aggregation and AHI, with elevated platelet distribution width and mean platelet volume (MPV) in OSA patients [74]. Consistent with this, reports highlight significant increases in adenosine 5′-diphosphate (ADP)-induced platelet aggregation in OSA patients [73]. Following CPAP therapy, OSA patients exhibit substantial reductions in platelet reactivity and MPV levels [75]. For patients on dual antiplatelet therapy combining aspirin and an oral P2Y_12_ inhibitor for ADP, CPAP appears to offer a distinct antiplatelet mechanism, reducing clopidogrel-induced antiplatelet effects and increasing high residual on-treatment platelet reactivity [76].

Despite variable effectiveness, CPAP is generally more cost-effective than dental devices, typical management procedures [77], and other emerging treatments. Although CPAP alternatives, including behavioral modifications, upper airway muscle exercises, oral appliances, and surgical interventions, have demonstrated partial effectiveness in alleviating sleep apnea symptoms [78], each method has limitations. Despite ongoing debates surrounding its efficacy, CPAP remains the standard treatment for most OSA patients. These considerations emphasize the need for engineering innovations in sleep apnea diagnostics and treatment, with substantial opportunity for further research and improved clinical understanding.

### 4.2. CSA Therapies

Therapeutic approaches for CSA are similarly limited, with positive airway pressure devices serving as the standard of care despite adherence challenges. Traditional CPAP fails to effectively address persistent ataxic central apnea patterns because it delivers constant pressure during both inhalation and exhalation [79]. Bi-level positive airway pressure provides non-chemical ventilatory drive stimulation in patients with ventilatory depression, but alternative solutions are being explored with varying degrees of effectiveness.

One such alternative is adaptive servo-ventilation (ASV), a newer form of positive airway pressure therapy designed specifically for CSA patients. ASV monitors breathing patterns in real-time and dynamically adjusts pressure based on individual apneic events during sleep [80]. Recent studies highlight ASV’s efficacy, particularly in cases of opioid-induced CSA [81]. However, caution is warranted as findings from the SERVE-HF clinical trial have contraindicated ASV because of an increased risk of sudden cardiac death in a subgroup of CSA patients with systolic HF [82]. This has tempered enthusiasm for broader ASV adoption despite its technical advantages.

The relationship between CSA treatment and HF outcomes is complex. Many CSA patients exhibit Cheyne–Stokes respiration, which may periodically produce CPAP-like effects that compensate for severe HF, rather than contribute to disease pathogenesis [63]. This raises concerns that CSA treatments could worsen HF in certain patients, emphasizing the need for individualized consideration before implementation [63]. While respiratory stimulants like acetazolamide and theophylline [83] have shown efficacy in improving respiration in CSA patients with HF, their use is coupled with an elevated risk of cardiac arrhythmias and sudden death [84]. Thus, it is still necessary to study alternative treatments for CSA.

## 5. Animal Models to Study Sleep Disordered Breathing

Animal models are invaluable tools for studying sleep-disordered breathing pathophysiology and developing pharmacological therapies. Various OSA models have been established to replicate different aspects of the disease [85], with the most common approaches focusing on intermittent hypoxia, mechanical airway obstruction, or anatomical narrowing.

The CIH model is the most widely used approach for studying cardiovascular consequences of OSA. This model simulates the cyclical hypoxia-reoxygenation pattern characteristic of clinical OSA, either through surgical interventions or using chambers that cyclically alter gas composition [86,87]. CIH exposure in rodents produces sustained sympathoexcitation, endothelial dysfunction and cardiovascular remodeling that correlate with the resistant hypertension and increased arrhythmia susceptibility observed in OSA patients [88]. CIH models have revealed numerous detrimental cardiovascular effects [89] and identified potential therapeutic targets. For example, chemogenetic activation of hypothalamic oxytocin neurons attenuates hypertension and cardiac dysfunction induced by CIH, highlighting neural mechanisms as promising therapeutic avenues [87]. Autonomic imbalance is one mechanistic explanation for cardiovascular morbidity in OSA patients, and clinical pharmacological strategies targeting this imbalance are being tested [90]. Beyond cardiovascular impacts, CIH models have also revealed significant neurocognitive effects. Recent evidence demonstrates that CIH impairs cognitive function and increases Alzheimer’s disease biomarkers in ovariectomized (estrogen-deficient) female rats, highlighting sex-specific vulnerabilities and potential links between OSA, estrogen deficiency, and dementia risk [91].

Alternative models focus on mechanical aspects of OSA. Negative pressure models create upper airway collapse through external pressure manipulation, while anatomical obstruction models induce spontaneous upper airway narrowing by artificially enlarging the tongue or soft palate through injection, replicating the anatomical narrowing observed in clinical OSA. However, OSA development involves multiple interacting factors, including anatomy, neuromuscular control, respiratory regulation and arousal thresholds [92]. Current models typically address only one or two of these factors, limiting their ability to capture the full disease complexity. Development of more comprehensive, multifaceted animal models that simultaneously incorporate multiple disease factors would provide a more comprehensive understanding of OSA pathophysiology and facilitate development of more effective OSA therapeutic strategies.

## 6. Recent Advancements in Treating Sleep Disordered Breathing

Given the limitations of conventional therapies for OSA and CSA, there has been growing interest in developing alternative approaches to enhance patient quality of life and reduce cardiovascular morbidity associated with sleep disorders. The clinical manifestations and consequences of sleep disordered breathing are diverse and vary considerably between individuals. This inherent disease heterogeneity means that no single treatment is universally effective, making identification of the most suitable therapy for each patient crucial to optimize outcomes while minimizing time, resource expenditure and patient discomfort. These challenges have driven innovation in device development and personalized treatment strategies, with epigenetics, nerve stimulation, and precision medicine emerging as promising avenues to advance sleep apnea management.

### 6.1. Epigenetics

OSA exhibits phenotypic variations influenced by genetic and environmental factors. While associations between OSA and genomic polymorphisms at numerous loci (for example, APOE, LPAR, PLEK, ARID1A, AHDC1, ARRB1) have been reported, genome-wide association studies have yielded inconsistent results [93,94,95]. Despite familial factors explaining 30–40% of OSA severity variability, only a few genetic polymorphisms, such as TNF-⍺-308G/A and serotonin 2A receptor-1438G/A have been independently linked to AHI [96,97,98,99]. Given these limitations of purely genetic approaches, recent research has focused on epigenetic mechanisms of disease susceptibility, including histone modifications, non-coding RNAs and DNA methylation. These mechanisms are influenced by environmental factors and play important roles in disease development [100], particularly in pediatric OSA, where epigenetic alterations may contribute to disease variability and progression [101].

Epigenetic modifications are heritable phenotypic changes that occur without alterations to DNA sequence that alter gene expression through multiple mechanisms. In response to hypoxic stimuli, DNA methylation and post-translational histone modifications can render the regions of gene promoters or enhancers more or less permissive to interaction with transcriptional machinery. Non-coding RNAs, including microRNAs (miRNAs) and long non-coding RNAs (lncRNAs) regulate gene expression by silencing or degrading targeted messenger RNAs. Together, these mechanisms modulate both the accessibility of genes for transcription factor binding and the rates of gene transcription.

Emerging evidence suggests that epigenetic changes play a role in OSA pathogenesis. In OSA patients, peripheral blood immune cells, endothelium and other end-organ tissues adapt to oxidative stress from CIH by modulating gene expression. For example, OSA patients exhibit decreased expression of SIRT1, a class III histone deacetylase (HDAC) (histone modifying enzyme) involved in eNOS regulation, cardiovascular function, stress resistance, and aging [102,103]. Conversely, OSA patients show elevated blood protein levels of HDAC2, linked to OSA-induced perturbations in visceral fat tissue depots, suggesting an association between histone-modifying enzymes and cardiovascular dysfunction in OSA [104]. In a pre-clinical CIH mouse model, aortic macrophages exhibited significant accumulation of the active histone mark H3K9ac, associated with pro-inflammatory and oxidative stress signaling pathways, including HIF-1, p53, NF-kB, tumor growth factor-β, forkhead box protein O4, and IL-6. In contrast, genes associated with the repressive histone mark H3K27me3 were linked to protective anti-inflammatory and glutathione redox pathways, including peroxisome proliferator-activated receptor/retinoid X receptor and liver X receptor/retinoid X receptor activation [105].

Epigenetic alterations represent potential biomarkers for early disease detection, prognosis prediction, and treatment response monitoring. Importantly, many epigenetic modifications are reversible, providing novel therapeutic targets. Several lymphomas and myelomas are currently treated with approved epigenetic therapies, including HDAC inhibitors such as Vorinostat, Romidepsin and Belinostat. Preclinical studies in cardiovascular health using HDAC inhibitors show promise for managing cardiac hypertrophy, HF, oxidative stress, hypertension, and cardiac fibrosis [106,107,108]. Notably, Givinostat, a clinical-stage HDAC inhibitor of catalytic activity, demonstrated efficacy in reversing impaired relaxation in two mouse models of HF with preserved ejection fraction [109]. These findings suggest both a mechanistic role for epigenetic regulation in OSA and potential for developing novel pharmacological agents to counteract OSA-related consequences, including oxidative stress, sympathetic activation, and low-grade inflammation [110].

In OSA, chronic intermittent hypoxia induces specific epigenetic alterations, including reduced SIRT1, increased HDAC2 activity, and accumulation of activating histone marks (e.g., H3K9ac) at the promoters of pro-inflammatory and oxidative stress genes (e.g., NF-κB, IL-6, TGF-β). This occurs along with suppression of antioxidant and metabolic pathways (such as PPAR/LXR signaling via H3K27me3), resulting in persistent transcriptional reprogramming in vascular and immune cells. These modifications promote endothelial dysfunction, vascular inflammation, oxidative stress, and maladaptive remodeling, ultimately manifesting as hypertension, atherosclerosis, and cardiac dysfunction in OSA.

### 6.2. Nerve Stimulation

**Figure 6:**
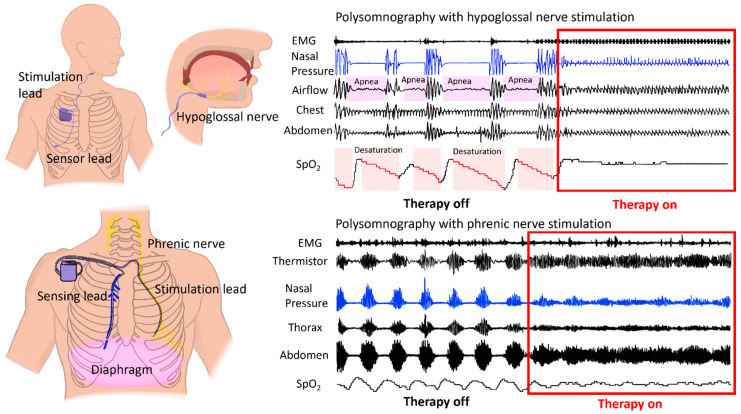
Comparison of hypoglossal and phrenic nerve stimulation therapies for sleep apnea. The diagrams illustrate the anatomical placement of stimulation leads for each therapy, and the physiological changes in the illustrated idealized polysomnography signals. The therapy “on” sections highlight improved respiratory parameters, including airflow, nasal pressure, and oxygen saturation (SpO2), compared to the therapy “off” conditions.

Two promising nerve stimulation approaches have been developed for sleep apnea treatment: hypoglossal nerve stimulation for OSA and phrenic nerve stimulation for CSA, each targeting different physiological mechanisms (Figure 6). While electrical stimulation for OSA treatment was first studied in the 1980s [111], recent technological advances have significantly refined these approaches. Early transcutaneous stimulation therapy was proposed as a simple alternative to CPAP, but its effectiveness has been variable depending on electrode placement and stimulation parameters [112].

Hypoglossal nerve stimulation has emerged as an effective therapeutic option for OSA [113] (Figure 6). During OSA, diminished genioglossus muscle tone causes tongue retraction that blocks airflow. Hypoglossal nerve stimulation prevents this retraction by activating tongue muscles to maintain airway patency [114]. The FDA-approved Inspire device uses this approach, where a pacemaker-like device is implanted in the chest to monitor breathing patterns and deliver targeted electrical impulses during sleep to the hypoglossal nerve using a stimulation lead. This stimulation promotes forward tongue movement, preventing airway obstruction. Clinical studies demonstrate that hypoglossal nerve stimulation effectively reduces AHI and oxygen desaturation index while improving patient outcomes [113,115,116].

However, hypoglossal nerve stimulation is not universally suitable for all OSA patients. It is typically considered for individuals who have failed CPAP therapy and meet specific qualification criteria. As with any surgical intervention, potential complications exist. Patient selection based on established criteria and individualized adjustment of stimulation parameters are essential for optimizing outcomes and represent important steps toward personalized OSA treatment [117].

Phrenic nerve stimulation is a newer approach for CSA that shows promise for patients with concurrent congestive HF [118]. The phrenic nerve innervates the diaphragm and coordinates breathing with respiratory muscles [119]. In comparison to CPAP, implantable systems for phrenic nerve stimulation directly address the dysregulated respiratory control characteristic of CSA without relying on patient compliance. These devices are surgically implanted in the right pectoral area with leads that stimulate the phrenic nerve to trigger appropriate breathing patterns (Figure 6). Incorporated feedback mechanisms ensure timely and adjustable stimulation aligned with the patient’s natural breathing patterns.

Studies have established the safety and efficacy of phrenic nerve stimulation in treating CSA [120]. Its effectiveness has been comparable to CPAP, demonstrating a 50% reduction in central AHI. However, long-term and larger-scale data are needed to address knowledge gaps. As additional data emerges, this approach holds potential as an effective management option for CSA, particularly in individuals diagnosed with congestive HF.

Beyond device-based nerve stimulation, recent advances in molecular neuromodulation offer additional therapeutic avenues. Oxytocin receptors are highly expressed in cardiac vagal neurons within the dorsal motor nucleus of the vagus and selective activation of these receptors produces rapid and sustained bradycardia. This oxytocinergic signaling pathway represents a promising future target for therapies aimed at restoring parasympathetic tone and cardiovascular stability in OSA patients [121].

### 6.3. Precision Medicine

Precision medicine offers a potentially transformative approach to OSA diagnosis and treatment by accounting for disease variability within the patient population and diverse treatment responses. Integrating gold-standard treatments like CPAP with contemporary precision medicine methods, including advanced bioinformatics and information technology, could enable development of patient-specific therapeutic strategies. For example, bioinformatic techniques have successfully identified differentially expressed miRNAs in OSA patients [122], and cardiac transcriptomic analyses in a neonatal sleep apnea model have revealed broad, sex-dependent changes in gene expression [123].

Circulating miRNAs have been identified as a potential biomarker for both OSA diagnosis and treatment monitoring. Following CPAP treatment, miRNA expression patterns in OSA patients align with those of non-OSA controls, suggesting their utility in assessing treatment response [124]. Three specific plasma miRNA profiles have been identified that accurately predict blood pressure responses to CPAP therapy [125]. Patient exhibiting these profiles experienced reduced blood pressure and decreased aldosterone-to-renin ratios after three months of CPAP intervention.

Additionally, 47 miRNAs altered by CPAP treatment have been identified. These advancements in miRNA profiling provide a foundation for individualized approaches to sleep apnea diagnosis and treatment, enabling adjustments tailored to patient-specific needs and facilitating nuanced responses to this complex disease.

Computational fluid dynamics (CFD) represents another promising integration of precision medicine into sleep apnea management. CFD models have successfully simulated multiple aspects of OSA, including upper airway structure [126], CPAP efficacy [127], oral appliance effects on respiratory parameters and surgical outcome predictions [128]. In one study [129], a CFD model evaluated OSA severity using flow-based parameters derived from CT-based 3D reconstructions of the upper airway. The model analyzed airflow and pressure distributions across different upper airway sections to predict collapse susceptibility during sleep. CT images acquired from awake patients were segmented to generate 3D anatomical models, which underwent CFD simulation. The researchers developed an adjusted pressure coefficient defined as the square root of the ratio of pressure changes to the velocity squared, which describes relative pressures throughout the flow field and identifies areas subject to collapse. This coefficient demonstrated stronger correlation with AHI than individual factors such as air velocity, wall shear stress, or pressure drop.

These findings suggest that anatomically based models augmented by CFD can provide detailed information about distinct upper airway components and help predict sleep apnea severity across varying patient phenotypes. Moreover, these models can be employed for both diagnostic purposes and treatment evaluation. While image-based CFD models currently face certain limitations, including assumptions of rigid wall boundaries, image acquisition from awake patients whose anatomy may differ during sleep and omission of smaller upper airway structures, they offer a cost-effective and highly adaptable approach to addressing the complex individual anatomy involved in sleep apnea. These computational approaches hold potential for providing mechanistic explanations of poorly understood aspects of OSA pathology and advancing personalized treatment strategies.

### 6.4. Artificial Intelligence

Artificial intelligence (AI) and machine learning (ML) are emerging tools in sleep apnea diagnosis and management. AI has the potential to address limitations in current diagnostic workflows and improve treatment personalization. Deep learning algorithms have been applied to PSG scoring and interpretation with high accuracy. In one study, a deep learning algorithm analyzed PSG data from 100 patients. It detected apnea/hypopnea events with high sensitivity and specificity across all OSA severities [130]. Another study used nasal respiration flow, SpO2, and ECG signals acquired during PSG to detect apnea/hypopnea events with 94% accuracy and categorize OSA severity with 99% accuracy [131]. These examples demonstrate the potential for enhancing the efficiency and accuracy of PSG event scoring. In addition to PSG scoring, ML algorithms have been applied to signals from wearable and portable devices for apnea event detection. ML approaches are also being used for OSA phenotyping and treatment response prediction including predicting CPAP adherence [132,133]. In this context, AI could help clinicians move from a generalized diagnostic model toward personalized approaches where patient-specific data guides therapies [134,135].

Despite the potential for AI to make significant improvements in sleep medicine, important considerations should be addressed before complete integration into clinical practice. The use of AI can be difficult to generalize because many learning models are developed in specific contexts. Laboratory equipment and workflows also vary between institutions. Another concern is AI being biased to training datasets as training data can underrepresent the wide range of patient racial, ethnic, age, sex, and socioeconomic status [136]. Future studies should prioritize external validation, prospective testing, transparent reporting, and evaluation of whether AI-assisted workflows meaningfully improve clinical outcomes. Regulatory considerations will also be important, especially for software as a “medical device” [137]. Because AI models can change over time and in different clinical settings, regulatory frameworks will need to address validation and transparency to ensure that AI technologies are used safely and appropriately. Ethical and legal challenges should also be addressed to create a safe and equitable healthcare system [138].

Overall, AI has a promising role in future sleep medicine, from diagnosis to disease management, but clinical implementation will require more diverse training datasets and rigorous external validation, and thoughtful workflows that preserve clinician oversight.

## 7. Conclusions

OSA and CSA are both complex diseases that drastically differ from patient to patient. Their impact extends beyond sleep disturbances, encompassing severe cardiovascular consequences and a noticeable decline in overall sleep quality, thus diminishing the overall quality of life for affected individuals. There has been a big push in recent years to uncover the nuanced mechanisms underpinning these diseases, aiming for a deeper understanding of how the disease pathologies manifest for the goal of developing more precise and effective targeted treatments. Significant advances in biomedical technologies support this important goal, highlighting new ways to treat sleep breathing disorders.

## Data Availability

No new data were created or analyzed in this study.
